# Periodontal therapy for patients before and after radiotherapy: A review of the literature and topics of interest for clinicians

**DOI:** 10.4317/medoral.22474

**Published:** 2018-09-28

**Authors:** Milena-Suemi Irie, Eduardo-Moura Mendes, Juliana-Simeão Borges, Luis-Gustavo-Gonzalez Osuna, Gustavo-Davi Rabelo, Priscilla-Barbosa-Ferreira Soares

**Affiliations:** 1 PhD student, Faculty of Dentistry, Federal University of Uberlândia, Uberlândia, Minas Gerais, Brazil; 2 Master student, Faculty of Dentistry, Federal University of Uberlândia, Uberlândia, Minas Gerais, Brazil; 3 Graduate student, Faculty of Dentistry, Federal University of Uberlândia, Uberlândia, Minas Gerais, Brazil; 4 Researcher, Faculty of Dentistry, Federal University of Uberlândia, Uberlândia, Minas Gerais, Brazil; 5 Professor of Periodontology Department, Faculty of Dentistry, Federal University of Uberlândia, Uberlândia, Minas Gerais, Brazil

## Abstract

**Background:**

To review and discuss important topics regarding periodontal treatment pre- and post-radiotherapy for head and neck cancer in human patients; to discuss the references for adequate techniques, the appropriate moment for tooth extractions and periodontal management; and to discuss the prevention of osteoradionecrosis.

**Material and Methods:**

Thirty-nine studies including original studies, randomized clinical trials (RCTs) and reviews were searched in online databases MEDLINE (PubMed) and the Cochrane library. No year of publication restriction was applied.

**Results:**

Language was restricted to English, and the following Medical Subject Heading terms were used: radiotherapy, radiation therapy and periodontal treatment. Studies regarding periodontal treatment and tooth extraction that involved clinical management of irradiated patients were selected.

**Conclusions:**

The treatment of periodontal diseases before radiotherapy is mainly required to avoid future dental extraction and to reduce the development of osteoradionecrosis. Periodontal treatment in irradiated patients mostly includes scaling and root planing, extraction of condemned teeth and topical and systemic antimicrobial therapy. Tooth removal should be planned at least 14 days before the first day of radiation treatment. Particular care and mouthwashes should be taken during and after radiation.

**Clinical Significance:**

The management of irradiated patients represents a challenge for health professionals, including dentists. It is important to establish recommendations for clinicians concerning dental and periodontal management in irradiated patients before, during and after treatment.

** Key words:**Head and neck cancer, radiotherapy, periodontal treatment, periodontitis.

## Introduction

Radiotherapy (RTX) is one of the treatment options for head and neck cancer, and it is also indicated as an adjuvant after tumour resection in association with chemotherapy or as palliative treatment for unresectable late-stage tumours (1). Management of irradiated patients represents a challenge for health professionals, including those working in the dentistry field. Many secondary effects related to RTX can occur in the oral cavity, including mucositis, xerostomia, loss of taste, trismus, progressive periodontal attachment loss, dental caries, soft tissue necrosis, and osteoradionecrosis (ORN). These effects can make the overall treatment and post-rehabilitation processes difficult (2).

In addition to the side effects described above, oral cavity morbidities related to RTX also include increased susceptibility to dental caries, tooth decay and periodontal disease (3,4). Patients irradiated in the head and neck region have increased risk for periodontal disease, because it usually is associated with hyposalivation and oral microbiome modification; moreover, periodontitis has been considered a trigger of ORN (5). At the microscopic level, oral keratinocytes revealed loss of proliferative capacity and increased production of proinflammatory cytokines in a radiation dose-dependent manner (6).

The effect of radiation on periodontal health is dose-dependent and is associated with poor periodontal health before radiotherapy initiation. The prevalence of periodontitis in adults is frequent and is likely to get worse with oncology treatment (4). The aggravation of periodontitis after radiotherapy may then require tooth extraction, which may result in ORN (5). The local effect on periodontal tissue when high dose fraction is used involves alterations in the cellularity, vascularity, and reduced healing/remodelling potential of the periodontium (7).

Recently, RTX has been applied through the use of three-dimensional (3D) irradiation techniques, such as 3D conformal radiation therapy (3DCRT) or intensity-modulated radiation therapy (IMRT). Both techniques have been shown to preserve adjacent tissues and provide a more precise assessment of high-irradiation and high-risk areas (8).

With the action of clinicians within dentistry, it is possible to propose a prevention-based approach, with control over the source of dental infection adapted to a patient’s situation before administering RTX. Pre-treatment assessment and management, accompanied with maintenance of oral hygiene, have already been shown to be effective methods of preventing oral and systemic complications following RTX treatment (8,9). Oral and periodontal examination is appropriate for all patients planning to receive head and neck RTX and for those who are to undergo medical procedures that will result in neutropenia. Oral and periodontal care must be applied before, during and after cancer therapy and requires knowledge of the characteristics of the neoplastic lesion, the oral manifestations related to impaired systemic condition, and the whole medical management of the disease, including the side effects of the treatment (5,7).

Thus, the aim of this study was to review and discuss important topics regarding periodontal treatment pre- and post-RTX for head and neck cancer in human patients. In addition, this study seeks to establish recommendations for clinicians concerning dental and periodontal management in irradiated patients before, during and after treatment. Lastly, this study includes a discussion of references for adequate techniques, the appropriate moment for tooth extractions and periodontal management, and the prevention of osteoradionecrosis.

## Material and Methods

Twenty four original studies, 1 randomized clinical trial (RCT) and 12 reviews were included, being the studies searched in online databases MEDLINE (PubMed) and the Cochrane library, all of them selected by means of the focus on the patient attendance and care. No year of publication restriction was applied. Language was restricted to English, and the following Medical Subject Heading terms were used: radiotherapy, radiation therapy and periodontal treatment. Studies regarding periodontal treatment and tooth extraction that involved clinical management of irradiated patients were selected, with an emphasis on updated guidelines and protocols.

## Review of Literature

- Pre-radiation procedures

• Clinical evaluation – what is important to consider?

Current recommendations indicate that irradiated patient examination and treatment should account for the cancer treatment. The clinician should examine the oral condition before, during, and following cancer therapy. Additionally, a lifelong commitment by the physician to promote preventative oral health management is recommended. Preventive maneuvers should be done in order to minimize the risk of worsening periodontitis, which could require tooth extraction with the associated risks of ORN (5). Periodontal attachment loss is greater on teeth located in irradiated sites. In planning pre-radiation treatment, the additional attachment loss over time that may affect the prognosis of the remaining teeth should be taken into consideration. The periodontal breakdown in xerostomic patients is comparable to patients suffering from Sjogren’s syndrome, though more significant periodontal destruction occurs in teeth within irradiated bone (7).

Tooth extraction recommendations defined before RTX should determine the right moment to perform this clinical procedure and which teeth should be extracted (10). Apical lesions indicated by apical periodontitis, periapical abscess and cysts are risk factors for the development of ORN (8). For instance, because most cases of ORN occur in the mandibular molar region, the teeth in this region that have periapical lesions may be extracted or receive root canal treatment (11).

• Tooth extraction and soft tissue management – reducing the risk of orn

The most followed guidelines and recommendations concerning tooth removal before radiation is based on the orientations of the German Society of Dental, Oral and Craniomandibular Sciences, which include the following: (1) periodontal probing depth equal or greater than 5 mm; (2) furcation involvement; (3) carious lesions that reach the pulp; (4) impacted and retained teeth; (5) large fillings, fractures, or significant occlusal wear; (6>) teeth positioned in a region of that is expected to receive more than 55 Gy; (7) teeth that are non-vital and without sufficient root canal filling; and (8) teeth that are painful, sensitive to percussion, or show apical radiolucency (8).

Teeth that cannot be preserved for a long time should be extracted before RTX, and strict dental management after treatment should be conducted. After RTX, dental treatment should be managed by a dentist in a continuous programme with topical fluoride administration and periodontal health management. It is important to emphasize that some patients develop ORN in the region where an intact tooth was within the radiation field, and this should be considered while following all patients who will be submitted to radiation in the head and neck region (10,11). In addition, it is also important to consider the periodontal status concerning not only tooth removal, but also the whole oral condition. Current evidence supports a link between periodontitis and oral mucositis associated with radiotherapy (12).

• Periodontal disease treatment

Periodontitis is a multi-factorial disease associated with the loss of supporting tissues, such as periodontal ligament and alveolar bone. The aim of periodontal therapy is to eliminate bacterial biofilm from the root surface (13). Significant improvements in clinical parameters were reported after non-surgical periodontal treatment (14).

Non-treated periodontal disease that exists pre-radiotherapy increases the risk of tooth extraction, and consequently, ORN. Analysis from a retrospective study revealed that patients with periodontal pockets > 6 mm were prone to develop ORN. The results of this study suggest that teeth with dubious prognosis that were maintained for prosthetic use may put patients at increased risk of developing ORN (15). Evidence has shown that patients with periodontal disease before radiotherapy are more susceptible to develop bone healing problems (4). Untreated periodontitis may also result in acute or chronic complications in patients undergoing radiotherapy (7). Because radiotherapy may aggravate periodontal disease or increase the risk of ORN, a meticulous evaluation of the patient’s periodontal condition by clinical and radiographic examination should be performed. Panoramic and periapical radiographs must be carried out initially. Periodontal charts, including probing depth, clinical attachment level, gingival recession, dental mobility, furcation involvement and bleeding on probing, must be performed on all teeth (4,16).

The periodontal status of a patient scheduled for radiotherapy is assessed by taking into account different criteria from other patients, with the main differences related to teeth extraction recommendation. Thus, to define the dental extraction procedure, the parameters of the radiotherapy course should be known, such as dose, time and the structures included within the field. Periodontal/dental/oral status must also be investigated in order to define a consistent treatment plan (5). Supragingival prophylaxis is recommended for all patients prior to radiation therapy. In cases of periodontal pockets ≥ 4 mm, subgingival scaling and root planning should be performed (17). Additionally, it is important to consider the treatment time and the number of sessions while avoiding RTX delay when periodontal treatment planning is indicated before the beginning of the radiation treatment.

- During radiation therapy

• Periodontal effects of radiotherapy 

Changes in the vascularity and cellularity of soft and hard tissues, salivary gland damage, and altered collagen synthesis are responsible for the clinical effects of radiation therapy (1). These alterations lead to a hypovascular, hypocellular, and hypoxic tissue (18). Thus, the remodelling ability of bone and soft tissue is compromised, and the risk of infection and necrosis is higher (7). Blood vessels in the periodontal ligament may result in widening of periodontal ligament space and destruction of the trabecular adjacent bone (19). Increased risk of periodontal disease and impaired bone remodelling and repair capacity are frequently observed (7). Gingival fluid and salivary flow are altered and could indicate a decrease in circulating immunoglobulins (20). If adequate oral hygiene is not established, periodontal destruction will likely occur.

Probing depth, clinical attachment level, gingival recession, plaque index and gingival bleeding on probing that was measured prior to irradiation and 6 to 8 months following radiotherapy verified that a greater loss of attachment occurs during this time compared to non-irradiated sites. No significant difference in plaque and bleeding on probing has been associated with these patients (21). A study conducted with a 2-year follow-up cohort included 56 patients treated with intensity-modulated radiation therapy (IMRT) who completed pre-radiation dental screening in order to eliminate oral foci in patients with head and neck cancer. The results revealed that twenty-four percent of dentate patients had progression of periodontal pocket depth (4-5 mm pockets deepened) and/or developed new periodontal pockets ≥ 4 mm after radiation (4). Gingival recession is another important clinical sign of periodontal disease progression in patients undergoing radiation directly on the periodontium (21), which requires increased radicular exposure in most of the patients. This situation may result in radiation caries and/or dental sensitivity.

- Post-radiation therapy

• Patient orientation and oral hygiene maintenance

After radiotherapy, biofilm reorganization is altered (22). Susceptibility to plaque accumulation plays a crucial role in disease progression in irradiated patients (23). Recent research has shown that treatment of head and neck cancer with IMRT with or without chemotherapy alters oral microflora, which in turn increases the incidence of opportunistic pathogens (24). Controlling plaque accumulation is essential to prevent periodontal pocket colonization in patients with decreased local defences and salivary changes (21). Patient instruction on oral hygiene maintenance before treatment is fundamental, and it is also necessary that patients avoid alcohol consumption and smoking (17).

Oral complication severity might decrease when oral hygiene protocols are performed. Use of a softer and smaller toothbrush and fluoride toothpaste should be recommended (25). Prior to radiotherapy treatment, the Modified Bass brushing technique, control of interdental plaque using dental floss or interdental brushes, and cleaning methods of the tongue must be explained to the patient (26).

Irradiated periodontium is more susceptible to the loss of attachment and to gingival recession (4). To prevent radicular caries and dentin hypersensitivity, the use of products containing fluoride is recommended. Neutral sodium fluoride gel (1.1%) should be applied for 5 minutes daily while the mouth is dry and saliva flow is reduced. This procedure can avoid “rampant” tooth destruction (25). During follow-up, dentate patient reductions in plaque scores (50% to 30%) and bleeding scores (30% to 10%) were observed in association with daily tooth brushing and the use of fluoride gel (4).

The ability of mouthwashes to prevent damage to the mechanical properties of irradiated enamel and dentin was tested. This study demonstrated that 0.05% sodium fluoride and 0.12% chlorhexidine prevented a decrease in tensile strength in irradiated enamel and dentin, respectively. Therefore, these substances can be used to reduce the side effects of radiotherapy treatment in irradiated teeth (27). When mechanical control of plaque is difficult, chemical plaque control with chlorhexidine 0.12% alcohol-free mouth rinses can avoid the occurrence of microbial infections and gingival inflammation and reduce the risk of caries (28).

• Supportive periodontal therapy

Patients treated with anti-neoplastic therapy had higher plaque indices (PI) and gingival indices (GI) than healthy patients. The use of fluoride products and chlorhexidine rinses are beneficial in patients after RTX (28). It is crucial to proceed with continuous attention to patients, as the detrimental effects of cancer treatment can affect their oral health throughout their lifetime (29).

Advances in cancer treatment have increased patient overall survival, though treatment usually requires aggressive management of oral toxicities related to the side effects of oncology therapies. To ensure long-term oral health and overall well-being, advances in oral care will need to reduce oral complications, which is unavoidable for these patients. The significant impact of long-term complications can be reduced through adequate prevention tasks. Prevention and management are best provided through multidisciplinary and integrated health care teams, including the dentist, in order to provide timely, coordinated treatment (30).

Hyposalivation and an increase in microorganisms after RTX associated with changes in diet and oral hygiene difficulties (mucositis, for example) increase the deve-lopment of dental caries and periodontal disease (31). The effect of radiotherapy on periodontal health is dose-dependent, and the impact of age and co-morbidity on radiation-related complications requires further investigation (5). Decreased vascularization of the periodontium leads to an enlargement of the periodontal ligament space. This event is associated with trauma and poor oral hygiene resulting in post-radiotherapy periodontal destruction (7). The most important late complication is ORN, which is characterized by an aseptic necrosis of the irradiated bone and loss of regenerative capacity of the osteogenic tissue. The soft tissue covering the bone becomes altered, and together with other stimuli, leads to lesions that do not heal (32).

Advances in radiotherapy techniques, such as the use of IMRT, allows high-dose irradiation of tumours with small volumes of exposed mandibular bone and has been observed to reduce the incidence to less than 5% (33,34). Additionally, the implementation of intra-oral devices can lower the dosage received by the tissues surrounding the tumour site. An intra-oral stent was effective in decreasing radiation dosage to healthy structures (35). In addition, preventive oral care decreases the incidence of ORN. Poor oral hygiene associated with local factors, such as maladaptive prostheses or post-RTX dental extractions, may raise the incidence of ORN to more than 25%. Poor oral health and local tumour progression may also increase the risk of ORN (34). There are several management protocols for treating ORN, such as conservative therapy with medication, ultrasound, hyperbaric oxygenation, low-level laser therapy and others (5). However, further prospective studies are needed to determine the long-term success of these protocols.

After IMRT for treatment of head and neck cancer, teeth can be preserved for longer time periods. This fact explains the increase in the depth of periodontal pockets, because preserved teeth remain in contact longer with periodontal pathogens. Although IMRT reduces the risk of xerostomia, a study with a one-year follow-up showed that opportunistic pathogens still increase over time (24). Oral and periodontal care must continue after radiotherapy and requires that the health professional understand cancer, medical management of the disease and its manifestations, and oral complications (7). Periodontal health maintenance programmes should provide important support to the irradiated patient. Maintenance of good oral hygiene in patients undergoing RTX may reduce the morbidity of known oral and periodontal side effects (1,19). Periodontal conditions among patients undergoing radiation therapy with or without chemotherapy in the head and neck region were evaluated. It was suggested that continuous periodontal management improves periodontal status and reduces plaque index, depth of probing, and bleeding, and maintains the insertion level in these patients (31).

Studies including case reports have indicated that periodontal surgery can be performed on select patients after radiotherapy. A careful evaluation of the previous RTX conditions must be performed, including whether the irradiation included the maxilla or mandible, the total dose, the fractionation and the source of radiation. Late tissue changes due to radiation should be evaluated to reduce the risk of osteoradionecrosis (5,10). Periodontitis and poor oral hygiene predispose the patient to mucositis (36,37). Patients with mucositis tend to have worsened periodontal condition (12). Early reversal of mucositis has been observed in patients who used triclosan when compared with patients that used conventional sodium bicarbonate rinses during and after radiotherapy; this likely occurred because the antimicrobial action of triclosan accelerates healing by controlling microbial flora, which delay wound healing. However, no efficacy of triclosan in radiotherapy-induced mucositis was confirmed (36).

Infectious diseases result in significant morbidity and mortality in cancer patients. During and after cancer therapy, patients may continue to experience some degree of compromised immunity and suboptimal lymphocyte function, which in turn facilitates the development of oral infections. Treatment of periodontal diseases includes scaling and root planning, tooth extraction, and topical and systemic antimicrobial therapy, usually with agents targeting oral anaerobic bacteria, such as penicillin, clindamycin, or metronidazole (38).

Radiation therapy increases oxidative damage in infected periodontal tissues. Recently, melatonin has been more frequently evaluated in research studies because of its anti-inflammatory, antioxidant and free radical scavenging properties. Melatonin use has been observed to significantly reduce regional alveolar bone resorption in rats. However, the data do not distinguish whether melatonin improved the effects of radiotherapy or of periodontitis alone (39).

- Final recommendations

**Figure 1:**
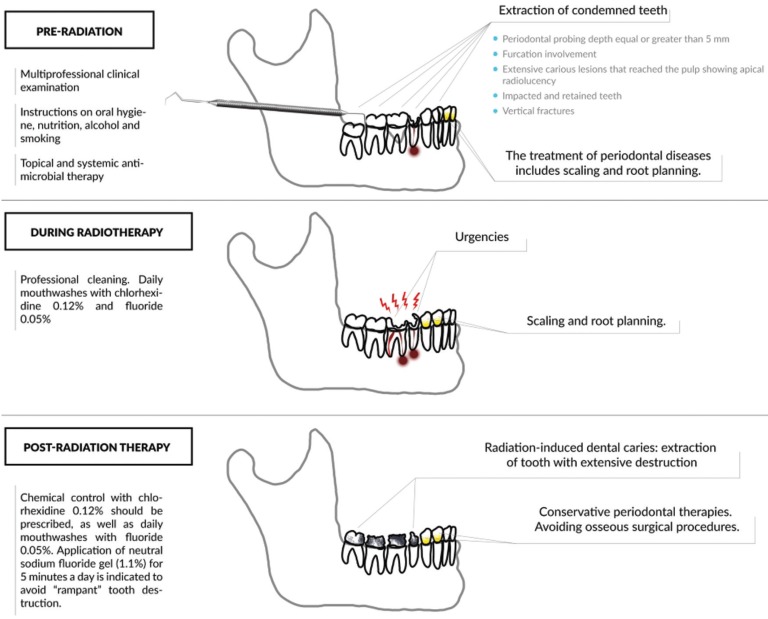
Periodontal treatment recommendations in irradiated patient.

Final recommendations to tend to irradiated patients should be based on procedures and clinical findings specific for the following time periods: pre-radiation, during and after radiation (Figure 1).

• Pre-radiation procedures

- The evaluation of the patient should be done by a multi-professional team that includes the dentist. The dentist should also help plan RTX with the implementation of intra-oral devices and/or other orientations concerning future rehabilitation plans.

- The clinical examination should assess the patient’s oral, periodontal and dental conditions for the purpose of addressing individual health problems. Clinical (periodontal chart) and radiographic (periapical and panoramic) examinations should be performed for pe-riodontal evaluation.

- Patients should be advised about the importance of maintaining oral hygiene and nutritional intake and should receive information about the deleterious effects of smoking and alcohol consumption. Oral hygiene instructions may include a description of the Modified Bass brushing technique, use of dental floss and interdental brushes, tongue cleaning methods and chemical plaque control.

- The treatment of periodontal diseases before RTX includes scaling and root planing, extraction of condemned teeth and topical and systemic antimicrobial therapy. Tooth removal should be planned at least 14 days before the first radiation treatment.

- For the prevention of ORN, the evaluation of the patient must be done by an experienced clinician considering the field to be irradiated, the total dose, the fractionation and the source of radiation, all of which will influence the indication for tooth extractions.

• During radiation therapy

- Prevention and cleaning of the oral cavity should be performed by a trained professional during RTX. When painful conditions make it difficult to brush the teeth (i.e., mucositis), other actions should be discussed and implemented. Chemical control with chlorhexidine 0.12% should be prescribed, as well as daily mouthwashes with fluoride 0.05%.

- Urgencies, scaling and root planing can be performed if platelet and white blood cell counts are within acceptable limits.

• Post-radiation therapy

- Post-RTX treatment involves prevention to reduce long-term complications, such as caries, periodontal diseases and osteoradionecrosis. Oral rehabilitation should be planned considering the loss of attachment that usually occurs in irradiated patients.

- Mechanical control of plaque/biofilm should be performed by the patient and by the professional.

- Chemical plaque control should include daily mouthwashes with fluoride 0.05% and chlorhexidine 0.12%.

- Periodontal therapies should be conservative, and bone surgical procedures should be avoided because of the risk of osteoradionecrosis. When these procedures are necessary, care should be taken, and tooth removal should be planned as a traumatic surgery, while switching to intra-oral sites for different tooth removal at different times should be considered.

- It is possible to perform periodontal surgery on irradiated bone, as in some cases this management can be tolerated more easily than extraction. This procedure should be discussed with the patient’s medical staff.
